# To stay or to move? Investigation on residents' migration intention under frequent secondary disasters in Wenchuan earthquake-stricken area

**DOI:** 10.3389/fpubh.2022.920233

**Published:** 2022-08-01

**Authors:** Huan Huang, Fan Wang, Yi Xiao, Yuan Li, Hui-Ling Zhou, Jing Chen

**Affiliations:** ^1^College of Business, Chengdu University of Technology, Chengdu, China; ^2^Yangtze River Economic Zone Research Institution of RUC, Yibin, China; ^3^Digital Hu Huanyong Line Research Institute, Chengdu University of Technology, Chengdu, China; ^4^Research Institute of Economics and Management, Southwestern University of Finance and Economics, Chengdu, China; ^5^College of Management Science, Chengdu University of Technology, Chengdu, China; ^6^College of Economics, Sichuan University, Chengdu, China; ^7^School of Accountancy, Tianfu College of SWUFE, Mianyang, China; ^8^School of Architectural and Engineering, Tianfu College of SWUFE, Mianyang, China

**Keywords:** secondary geological disaster, Wenchuan earthquake disaster area, migration intention, linear probability model, profit-seeking and harm-avoiding

## Abstract

The deterioration of the living environment caused by the earthquake is the main migration motivation of residents in the area of the secondary earthquake disaster, and their migration intention is one of the most important factors affecting residents' happiness. This paper uses 957 effective survey samples from 12 secondary geological disaster areas after the Wenchuan earthquake to research the migration intention of residents and its influencing factors. It can be found that 45.2% of residents are willing to migrate, which means they have an instinctive reaction to profit-seeking and harm-avoiding, but it has not become a realistic choice. Investigation facts and research results show that the instinctive response of profit-seeking and harm-avoiding drives residents to make different choices. The migration of residents in areas where secondary geological disasters occur is affected not only by disasters such as debris flow, landslides, and collapse, but also by many factors such as life convenience, family income, expectations for future life, gender, education level, psychological feeling. The improved life and the optimization of the economic conditions brought about by the success of post-disaster reconstruction have made the vast majority of people more confident in the future of the disaster-stricken areas, which made most people choose to stay in those areas. This paper will provide policy suggestions for residents' migration and the reconstruction of the local social governance system in secondary geological disaster areas, which is helpful to improve ecological livability and residents' happiness in the Wenchuan earthquake-stricken area.

## Introduction

The 2008 Wenchuan earthquake has caused severe economic losses and ecological damage to Sichuan Province, China, and the frequent secondary geological disasters have directly threatened the lives and property safety of residents in the disaster areas (1). The earthquake brought severe losses to Wenchuan County, about 96,000 people were killed and about 288,000 injured (2), and caused more than 56,000 landslides and slope failure (3), rockfall and debris flow in the form of about 20,000 people were killed (4). At the same time, the road network caused huge damage and economic losses (5). The official direct economic loss was 845 billion yuan (124 billion US dollars), which also caused a huge indirect economic loss (6–9).

Profit-seeking (PS) and harm-avoiding (HA) is an instinctive behavior in people's infancy. Adam Smith's “Economic man hypothesis” has clearly pointed out that driven by economic incentives, people will strive for their best interests and be compensated (10). However, as for the long-term research on the Wenchuan earthquake-stricken area, the research team of this paper has found that in the secondary geological disaster area, although residents' personal and property safety is greatly threatened, there was not much emigration of the local population. The secondary geological disaster frequently occurred in the Wenchuan earthquake-stricken area. But the registered population of Wenchuan County dropped from 104,131 in 2008 to 95,891 in 2017, a decrease of 7.9% overall, and the change was not significant.

According to the information disclosed by the People's Government of Wenchuan County, the Caopo Town in Wenchuan County being canceled due to secondary geological disasters is very typical. Natural hazards such as mudslides and mountain collapses have occurred in Caopo Town. Landslides occurred in 80% of the mountains, 65% of the cultivated land was damaged to varying degrees, 100% of the rural population suffered disasters, and the ecological environment was severely damaged. Among the 286 new geological disasters, more than 140 kilometers of roads were damaged, 17 bridges were destroyed, and infrastructure such as water, electricity, and communications were damaged. According to experts' assessment, Caopo Town no longer has living conditions. The government decided to rebuild Guojiaba off-site in Shuimo Town, and the original Caopo Town was merged into Mianlou Town. However, a phenomenon was discovered in the survey of the previous Caopo Town. Many residents of the original Caopo Town who moved out have returned to their original sites and only returned to resettlement houses during the high incidence of secondary geological disasters in summer.

Then, in these areas with frequent secondary geological disasters, are residents willing to migrate? What are the factors affecting residents' willingness to relocate? How do these residents choose PS and HA? To get answers to these questions, the research team of this paper went to Wenchuan earthquake-stricken area to conduct field investigations. The following 12 towns were investigated: Yingxiu, Xuankou, Weizhou, Shuimo, Miansi, Yinxing, Yanmen, Keku, Qushan, Chenjiaba, Xinzhen, and Qingping. And from the perspective of the affected people, this paper studies the changes and influencing factors of residents' willingness to migrate from the Wenchuan earthquake-stricken area where secondary geological disasters frequently occur.

Residents' migration has become a global issue of concern. Capital, knowledge, information, and other factors will flow together with the population movement, so the population distribution pattern greatly impacts regional development (11). The main research on residents' migration focuses on the analysis of population migration factors and the analysis of regional population structure (12).

The character of the attitude-behavior relationship pervades human decision-making theory and research. The underlying framework for migration decision research includes the following general components: background factors (both personal and structural), perceptions of place utility, intentions to move, and migration behavior (13). According to the theory of reasoned action proposed by Ajzen and Fishbein (14), migration intention remains the dominant determinant of migration behavior. Regarding residents' willingness to migrate from the frequent secondary geological disasters area, some scholars took the Longmenshan fault zone as the research object for field investigation. Field surveys show families in the Longmenshan area often choose to recover from their original residence (15). Their research shows that residents prefer to take disaster risks rather than migrate. Also reluctant to move are the Louisiana residents of Terrebonne Parish, who would rather endure rising waters, hurricanes, and tropical storms than live elsewhere (16). The study of Miyagi Prefecture, a site affected by the Great East Japan Earthquake and Tsunami, concluded that the migration was forced, and the scholars examined the main factors that led people to migrate (17). Also arguing that migration is forced, Thomas and Benjamin assess the policies and mechanisms in Caribbean and Pacific small island developing states (SIDS) to deal with climate-induced migration and displacement (18).

Existing research on regional population structure showed that the accumulation of population migration data affects the age structure of the region, and it is an integral part of the population composition of an area (19–22). Network analysis method [(23–26)], multiple regression analysis [Huang et al., 2019, (27–30)], and population dynamics models (31) are often used to analyze regional population migration.

In the past 10 years, many scholars have done a lot of research on the Wenchuan earthquake (32). The occurrence of the Wenchuan earthquake not only caused a huge loss, but also brought a series of secondary geological disasters, including soil and debris flow, rockfall, landslide, the formation of barrier lake, etc. (33–35). These secondary disasters resulted in 87,419 deaths or missing persons and 374,176 injuries (33). Research results showed that the Wenchuan earthquake particularly impacted subsequent rainfall-induced debris flows [(36–38)]. The massive soil damage and debris become the material source of subsequent debris flow (39), which damages the stability of the surrounding mountains and brings long-term geological disasters to the Wenchuan earthquake-stricken area (40). It is expected that such tragedies will continue to occur in the future, resulting in substantial human casualties and economic losses in the Wenchuan earthquake-stricken area (40, 41). Faced with these problems, exploring migration willingness during the reconstruction period after the Wenchuan earthquake is much more significant (42).

The earthquake causes changes in the geological environment and induces the occurrence of secondary geological disasters, which will inevitably cause damage to the socio-economic system. Resident migration is an effective adaptation to the risks of secondary geological disasters (43). However, the actual situation is very different from the research assumptions. The migration caused by long-term environmental change is more complex than sudden natural hazards (44).

In summary, current research has focused on population migration in sudden major disasters and population migration due to urbanization. Moreover, the scope of research is broad, mainly studying population migration activities worldwide or analyzing population migration in provincial areas. Little literature has explored the micro-level population migration behavior and influencing factors. There is still a lack of research on the migration willingness of residents from areas where secondary disasters occur in the post-reconstruction period and its influencing factors.

**Figure 1:**
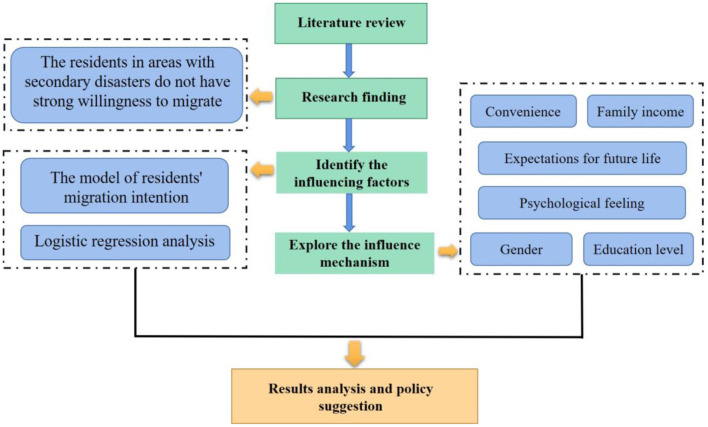
The logical framework.

This paper hopes to deeply analyze the migration intention of residents in secondary disaster-prone areas based on the field survey data conducted by the research team in the 10th year after the Wenchuan earthquake. By constructing an index system and applying the LPM analysis method, the research team studies the relocation intention of residents from Wenchuan earthquake secondary disaster-stricken areas and its influencing factors. The effects of each influencing factor on the migration decisions of residents from Wenchuan earthquake-stricken areas exposed to secondary geological hazards are analyzed in depth. The contribution of this paper compared to other studies lies in the following three aspects. Firstly, the research data, which took several months, reveals the current perceptions of residents in Wenchuan earthquake-affected areas about migrating out of secondary geological hazard-prone regions. Secondly, the first-hand microscopic data enriches the research scope and theoretical system of population migration. Thirdly, this paper provides insights into the factors affecting residents' migration in Wenchuan earthquake-stricken areas, which can be targeted to offer policy recommendations to the migration of residents and the reconstruction of social governance systems in secondary geological disaster areas. In conclusion, the research in this paper contributes to improving the ecological livability and residents' happiness in Wenchuan earthquake-stricken areas, and the starting point and level of the study are profoundly innovative. The theoretical model proposed in this paper is given in Figure 1.

## Data and methodology

### Research variables

The dependent variable in this study is “willingness to migrate.” It studies the willingness of residents to move to other places when there are opportunities. The categories are very strong, relatively strong, general, not very strong, and almost no one wants to move. In order to facilitate research, the first three categories are simplified as “move,” and the latter two categories are simplified as “stay,” so “willingness to migrate” is a dichotomous variable.

The selection of variables is integrated through reference to the actual research situation and combined with literature studies. Regarding the influencing factors of migration intention, whether rural residents settle down or not is mainly affected by social relations (45, 46). And for urban residents, education level, age, income, employment status, and occupation type are more important (47–50). Meanwhile, influenced by China's household registration system, rural homestead also affects the willingness of residents to transfer their registered permanent residence to a certain extent (51). From a macro perspective, the two main factors affecting population migration in China in the past decade are the employment environment and the livable environment (52–54). As seen in the existing research, it is common that social relations, education, employment status, age, income, and livable environment are the major factors affecting population migration. Following scientificity, rationality, and availability principles, six influencing factors (namely gender, education level, family income, life convenience, expectations for future life, and psychological feeling) are finally selected in this paper (Tables 1, 2).

**Table 1:** Variable selection.

**Variable name**	**Variable connotation**
Life convenience	It describes the distance between the residents' current residence and the supermarket, hospital, school, department store, railway station, and bus station. Long-distance will increase the cost of residents, and they are more likely to choose to migrate.
Family income	It measures residents' overall economic strength and financial and material resources in areas where secondary geological disasters occur, significantly impacting their willingness to migrate.
Expectations for future life	It describes the expectation and confidence of residents living in the current place of residence for future life. If they are full of confidence and hope, they are less likely to choose to migrate, and vice versa.
Gender	Different genders have different perceptions of current residence and living conditions, further affecting the intention to migrate.
Education level	It describes the choice of migration for different education levels. The education level will affect residents' thinking model and risk aversion awareness.
Psychological feeling	It includes the residents' feelings toward the local area, whether they feel at home in the residential community, the trust in neighbor relations, whether the neighbors get along well, and whether they are part of a neighborhood.

**Table 2:** Variable description.

	**Minimum**	**Maximum**	**Mean**	**Standard deviation**	**Variable definitions and assignments**
Willing to migrate	0	1	0.45	0.498	0 = No:l = Yes
Life convenience	1	5	2.81	0.974	1 = Very close, 2 = Relatively close, 3 = General, 4 = Relatively far, 5 = Far away
Family income	1	5	2.57	0.887	1 = Significantly higher than before the disaster, 2 = Some improvement, 3 = Almost, 4 = A certain drop, 5 = The economic situation has dropped a lot
Expectations for future life	1	5	1.95	0.855	1 = Hopeful, 2 = More promising, 3 = General, 4 = More pessimistic, 5 = Very pessimistic
Gender	1	2	1.56	0.496	1 = Male, 2 = Female
Education level	1	5	2.26	0.823	1 = Illiteracy, 2 = Primary school, 3 = Middle school, 4 = Junior college/University, 5 = Master degree and above
Psychological feeling	1	5	2.01	0.577	1 = Very affectionate, 2 = More affectionate, 3 = General, 4 = Not very emotional, 5 = No emotion

### Data sources

In this study, the areas under investigation were: Yingxiu, Xuankou, Weizhou, Shuimo, Miansi, Yinxing, Yanmen, Keku, Qushan, Chenjiaba, Xinzhen, and Qingping. The survey period is from January to August 2017, the 10th year after the 2008 Wenchuan earthquake. The questionnaire is divided into two forms: in-home interview and centralized location. Residents aged between 20 and 80 are randomly selected to fill in the questionnaire. For illiterate residents, the surveyors have filled out questionnaires according to the respondents' real intentions. In order to ensure the reliability of the questionnaire, the questionnaire is designed by referring to numerous pieces of literature on post-disaster reconstruction and people's willingness to migrate, and experts in these fields are consulted. The questionnaire items are screened to reduce the bias appropriately, and the questionnaire has been revised several times to get the final questionnaire. At the same time, this paper has added a questionnaire validity test question in the behavioral question section and collected additional questions on respondents' gender, marital status, family size, and area of residence as important supplementary conditions to judge the validity of the questionnaire. One thousand questionnaires have been sent out in this survey, and 957 have been recovered. The sample structure is shown in Table 3.

**Table 3:** Composition of samples.

	**Variable**	**Frequency**
Gender	Male	44%
	Female	56%
Marital status	Married	90.60%
	Spinsterhood	9.40%
Age distribution	20–31 years old	23%
	32–60 years old	48%
	61–80 years old	29%
The highest level of education	Primary school	16.3%
	Junior high school	45%
	High school or technical school	32%
	College degree or below	4.7%
	Master or above	3%
Physical condition	Good	63.80%
	Ordinary	24.80%
	Below the average	11.50%
Nationality	Ethnic Han	43.30%
	National minority	56.70%
Registered permanent residence	Village	75%
	Cities and towns	25%
Monthly household income of rural residents	3,000 yuan of the following	46.10%
	3,001–4,000 yuan	35.30%
	More than 4,001 yuan	18.60%
Monthly household income of urban residents	3,000 yuan of the following	43.30%
	3,001–4,000 yuan	23.30%
	More than 4,001 yuan	33.40%

**Figure 2:**
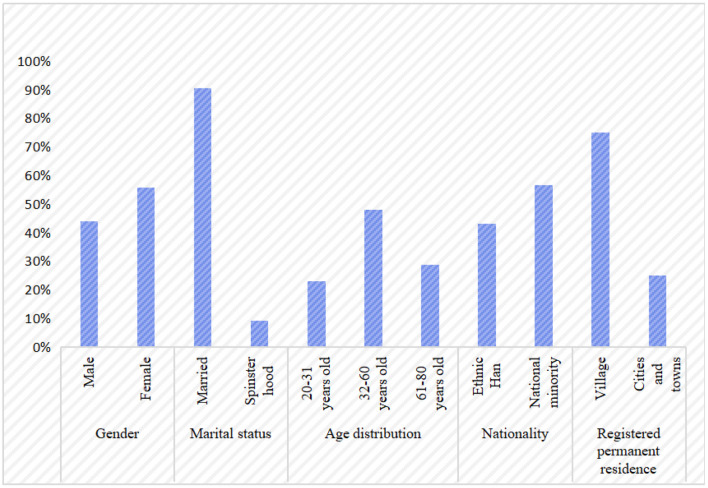
Basic information of residents in those valid samples.

According to Table 3 and Figure 2 the proportion of males and females in the valid sample is 44: 56, 90.6% married and 9.4% unmarried. The age of the total sample is mainly distributed in the 32–60 years old and over 60 years old, accounting for 48 and 29%, respectively. Among the interviewees, 56.7 percent are ethnic minorities, and 43.3 percent are of the Han nationality. Seventy-five percent of the interviewees are rural, and 25% are urban. The proportion of urban households with a monthly income of <3,000 yuan, 3,001–4,000 yuan, and more than 4 001 yuan is 43.3, 23.3, and 33.4%, respectively, and that of rural households is 46.1, 35.3, and 18.6%. According to the data, there is a big gap between rural and urban income. The highest proportion of respondents with a junior high school education is 45 percent.

### Research methodology

Since the respondents' willingness to migrate is divided into two categories, the dependent variable of the model is dichotomous. The logistic regression model is the most common method when studying the dependent variable as a dichotomous variable. It uses a logistic function to realize probability estimation and explore the relationship between variables (55). However, a key problem has not been solved: the coefficients of the Logit models are difficult to compare directly with each other.

In order to solve the problem, a linear probability model (LPM) can be considered as an alternative to the logistic regression model. For example, Woodridge's analysis of labor market participation data of married women used the LPM, Logit model, and Probit model, respectively, and the findings of the three models were found to be consistent (56). Karlson's simulated data analysis also showed that the LPM coefficient comparisons were closer to the actual differences than the simple direct comparison of Logit coefficients (57). The Logit and Probit models are used to study the problem that the dependent variable is dichotomous. The difference between them is that the conditional probability of the Logit model approaches 0 or 1 at a slower speed than that of the Probit model. Compared with LPM, although these two models can describe the nonlinearity of the total regression function from the probability, it is relatively more challenging to estimate the model and explain the regression coefficient. Therefore, the LPM of the dichotomous dependent variable is adopted in this paper to establish the model of residents' migration intention in the secondary geological disaster area.

According to the LPM, the quantified value of the dependent variable in this paper is 0 when the residents in the area where the secondary geological disaster occurs are willing to migrate, and 1 when the residents in the place where the secondary geological disaster occurs are unwilling to relocate. The construction model is as follows:


(1) $$\begin{array}{l} migration\;=\;\beta_{0}\;+\;\beta_{1}convenience\;+\;\beta_{2}income\;+\;\beta_{3}expectation\;+\;\beta_{4}gender\;+\;\beta_{5}education\;+\;\beta_{6}psychological\;+\;\mu \end{array}$$

Where “*migration*” denotes the dependent variable, a value of 0 indicates that residents are willing to move. The word “*convenience*” denotes the convenience of living, “*income*” denotes household economic income, “*expectation*” denotes expectations for future life, “*gender*” denotes gender, “*education*” denotes educational attainment, and “*psychological*” denotes psychological feelings. And *μ* denotes the error term, *β*_0_ is the intercept, *β*_1_, *β*_2_, *β*_3_, *β*_4_, *β*_5_, and *β*_6_ are the regression coefficients of the corresponding independent variables, respectively.

The descriptive statistical analysis is used to describe the phenomenon of residents' willingness to migrate from the areas affected by secondary geological disasters after the Wenchuan earthquake. In Table 4, it can be found that 524 people are willing to emigrate, accounting for 54.8% of the 957 valid questionnaires, while 433 people do not want to migrate, accounting for 45.2%. And then, LPM (willingness to migrate is 0, reluctance to migrate is 1) is used to study the influencing factors of desire to migrate or relocate. In-depth analysis of the factors that affect the migration willingness of residents in the Wenchuan earthquake-stricken area.

**Table 4:** Residents' willingness to migrate from the secondary disaster area.

**Frequency**	**Percentage**	**Effective percentage**	**Cumulative percentage**
Effective	0	524	54.8	54.8	Effective
	1	433	45.2	45.2	
	Total	957	100.0	100.0	

## Results

The “willingness to migrate” factor of residents in the secondary geological disaster area is the dependent variable, and key factors affecting residents' willingness to migrate are taken as the independent variable. The LPM (willingness to migrate as “0” and reluctance to migrate as “1”) is adopted to explore the influencing factors of residents' migration behavior. The results calculated by the software Stata15 are shown in Table 5.

**Table 5:** Variables and willingness of residents affected to migration.

**Y**	**Coef.**	**Std. Err.**	**t**	**P > | t |**	**[95% Conf. Interval]**	
Life convenience	0.073	0.014	5.22	0.000	0.045	0.100
Family income	0.392	0.018	2.15	0.031	0.003	0.075
Expectations for future life	0.074	0.019	3.88	0.000	0.036	0.111
Gender	0.062	0.032	1.96	0.050	−0.000	0.124
Education level	0.064	0.019	3.32	0.001	0.026	0.103
Psychological feeling	−0.059	0.018	−3.30	0.001	−0.093	−0.024
-cons	−0.109	0.942	−1.16	0.248	−0.294	0.076

It can be found that five variables that are significantly related to migration intentions, involving life convenience, family income, expectations for future life, gender, and education level, which are positively related to the dependent variables. And they have passed the significance test at the 5% level. Among them, life convenience, family income, expectations for future life, and education level are positively correlated with the willingness to move. The factor, psychological feeling, is negatively correlated with willingness to migrate. Regarding gender, the questionnaire survey shows that females are more willing to migrate.

Under the influence of the principle of PS, people are more willing to live in places with high living convenience. According to Table 5, the regression coefficient for the variable of living convenience is 0.073, which indicates that the convenience of living has a relatively important effect on the willingness to migrate. In the case of low-income family economic conditions and greater pessimism about the future, people will move elsewhere and seek new interpersonal relationships to get rid of the miserable life and obtain more development opportunities.

**Figure 3:**
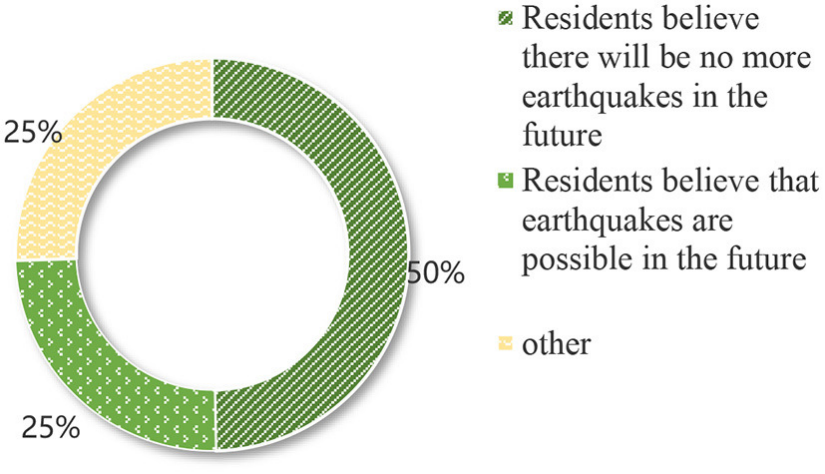
Residents' views on the prospect of an earthquake.

**Figure 4:**
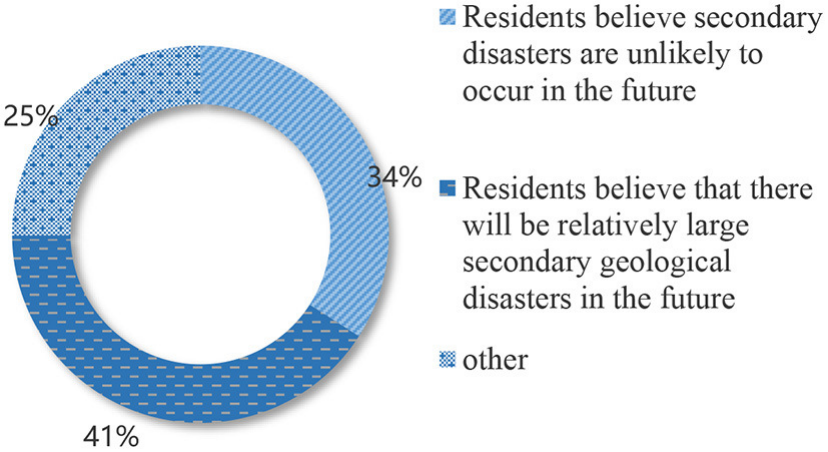
Residents' views on secondary geological disasters in the future.

As seen in Table 5, family economic conditions and expectations for future life significantly impact willingness to move. In particular, the regression coefficient of family income is as high as 0.392, indicating that family income plays a decisive role in their willingness to migrate. It is especially evident among females and people with higher education. According to the survey, 97% of residents are optimistic about the future of life, and only 3% are pessimistic. The survey shows that 49.7% of residents who have suffered earthquakes and live-in areas with frequent secondary geological disasters believe that there will be no more earthquakes in the future, and 34.1% believe that secondary geological disasters will be unlikely to occur. And 24.8% of residents consider that an earthquake might happen in the future, and 40.8% agree that a relatively large secondary geological disaster would occur in the future (as shown in Figures 3, 4). It indicates that residents in the Wenchuan earthquake secondary geological disaster area are optimistic about their future.

In the 12 villages and towns surveyed in this study, most of the males are migrant workers, and the females are raising children and providing for the elderly at home, while also undertaking farm work. These females suffer from the threat of secondary geological disasters and many inconveniences caused by poor living standards. At the same time, females care about the growth environment of their children by nature. Therefore, females are more willing to migrate to new places with good living conditions and high quality of life.

The proportions of respondents with illiteracy, elementary school, middle school, university, master's degree, and above who choose to migrate are 44.3, 69, 99.3, 100, and 100%, respectively, showing an increasing trend. The regression results in Table 5 also show that education level positively contributes to the willingness to migrate, with a regression coefficient of 0.064. The reason may be that the higher the education level, the more open-minded, and they will have stronger survivability and adaptability. Therefore, they are more willing to migrate to other places where their lives are more stable, avoiding risks and hoping to gain more space for development.

Psychological feeling and willingness to migrate are inversely related, and the regression coefficient for psychological feelings was −0.059 according to the regression results in Table 5. It shows that even if they have deep feelings for their hometown, facing frequent secondary geological disasters, residents are still willing to migrate under the influence of the principle of HA.

The geographic location of the districts can affect the risk of getting hit by natural hazards and their corresponding magnitudes. Therefore, the research team has added district fixed effects to study the willingness to migrate from different regions. The regression results based on Stata15 are shown in Table 6.

**Table 6:** Variables and willingness of residents affected to migration with district fixed effects.

**Y**	**Coef.**	**Std. Err.**	**t**	**P >| t |**	**[95% Conf. Interval]**	
Life convenience	0.064	0.014	4.70	0.000	0.037	0.091
Family income	0.046	0.018	2.63	0.009	0.012	0.080
Expectations for future life	0.019	0.019	0.98	0.328	−0.193	0.058
Gender	0.071	0.030	2.35	0.019	0.012	0.131
Education level	0.048	0.019	2.54	0.011	0.011	0.086
Psychological feeling	−0.065	0.018	−3.75	0.000	−0.098	−0.031
CODE						
Beichuan	0.126	0.039	3.24	0.001	0.050	0.202
Maoxian	0.022	0.076	0.29	0.775	−0.128	0.171
Mianzhu	0.486	0.056	8.74	0.000	0.377	0.600
-cons	−0.037	0.966	−0.38	0.707	−0.226	0.153

In Table 6, CODE represents the surveyed areas. The 12 towns surveyed are divided into four counties by administrative division: Wenchuan, Beichuan, Maoxian, and Mianzhu. And they are added to the regression as dummy variables. From Table 6, Beichuan and Mianzhu passed the *p*-value <0.05 and passed the significance test, while Maoxian failed the test. It indicates that the willingness to migrate is more significant in Beichuan and Mianzhu residents compared to Wenchuan, while Maoxian is less significant than Wenchuan.

However, what choice do the residents make in those areas where the secondary geological disaster occurred in the actual situation? The questionnaire results show that although the survey areas have experienced many geological disasters, 55.9% of the sample population still does not show a strong willingness to move out. Therefore, it is apparent that facing the unfavorable conditions of frequent secondary geological disasters, most residents in the disaster areas have not made the inevitable choice between PS and HA.

## Discussion

PS and HA are human instincts that have been manifested in infancy. It instinctively guides people to conduct cognitive reasoning and make judgments and behavioral choices (58). In the secondary geological disaster area, not all residents have a strong willingness to migrate to the safer, more economically developed, and more convenient regions. Isn't these residents' behavior to PS and HA? The facts of the survey suggest that the answer is not. Out of instinct, the reluctance to migrate remains partly a matter of PS and HA. The reason is that in the process of PS and HA, residents are affected by many factors such as life convenience, family income, expectations for future life, gender, education level, and psychological feeling.

- Life convenience. With the post-disaster recovery and reconstruction, many infrastructure facilities have been constructed, and measures to facilitate the people have been carried out (59). The living convenience of residents in the Wenchuan earthquake disaster area has far exceeded that before the earthquake (60). The lives of residents in the Wenchuan earthquake-stricken area are becoming more convenient, and the corresponding costs are lower, which objectively reduce the residents' willingness to migrate.
- Family income. The visible income is an important manifestation of PS. Although local geological disasters often occur, there are still certain rules to follow. Thus, residents' willingness to migrate is reduced. First, people with higher family economic income have a higher opportunity cost and higher cost of migration, so they are less willing to migrate (61). Second, it is generally believed that the higher the residents' income, the stronger the ability to migrate (62). These people are more likely to migrate to economically developed areas with more employment opportunities and no secondary geological disasters. A strong ability to survive does not mean a strong willingness to migrate. Field investigations show that most residents in the 12 towns in the secondary geological disaster areas chose to go out to work. Their income sources are more diversified than those of families whose income is mainly from farmland. The residents living in rural areas can obtain income from agricultural operations and higher income from non-agricultural work. Therefore, migration intention is insufficient, and their consciousness of PS and HA is correspondingly weak.
- Expectations for future life. Objectively, the willingness to migrate is lower due to the residents generally being optimistic about the future. From a social psychology perspective, no matter what people's considerations and goals are, people will experience complex psychological activities (63). Generally, residents believe that the greater the possibility of secondary geological disasters in the future, the more pessimistic they will be about their future life, and their willingness to migrate will be more robust (64). However, this is not the case. Instead, these people are hopeful about the future. Above all, with the continuous development of the national economy and the advancement of targeted poverty alleviation, the current development environment is trending for the better, making people full of confidence in their future life. In addition, the government attaches importance to the construction of infrastructure in areas with frequent geological disasters, and the building of communities has improved, making people's lives more colorful. Finally, residents believe in their ability to create a better future and are full of hope for life. Residents' inner understanding of society and their optimistic expectations dilute their willingness to migrate and dilute residents' interests and HA.
- Gender. In the areas of secondary geological disasters, males (especially middle-aged and elderly males) tend to live locally, and their migration intention is weaker than that of females. On the one hand, many males in the areas surveyed are migrant workers. They don't have the same life pressures as females and are not directly exposed to the threat of secondary geological disasters. On the other hand, males do not suffer the inconvenience brought by the poor local living standards like females at home. Therefore, their willingness to migrate to places with fewer secondary geological disasters, good conditions, and improved quality of life is not as strong as that of females. Secondly, most interviewees are middle-aged and elderly with deep feelings for their hometown. Even in the secondary geological disaster area, they have a weak awareness of migration or relocation.
- Education Level. Less-educated residents in the disaster zone are less willing to migrate. When the educational level drops by one unit, the probability of intention to migrate decreases by 1.25 times, because the less educated the residents are, the less open-minded they are, and the less able they are to survive and adapt to the unfamiliar environment (65). Therefore, it is difficult for them to make migration decisions. Secondly, due to the low level of education, the quality of their labor force is far from matching the market demand. If these people migrate to economically developed areas, they will lose the initiative of self-reliance. Finally, the lower the educational background, the less understanding of the harm caused by secondary geological disasters, the weaker the residents' awareness of risk avoidance, and the lower their ability and awareness of PS and HA.
- Psychological feeling. Psychological affection has an important influence on migration intention and PS and HA (66). Firstly, the residents' psychological needs and psychological endurance cannot be ignored. During the investigation, it is found that most of the residents have deep feelings for their hometown. The friendly neighborhood and environment, as well as the experience of disasters and sharing weal and woe, caused many residents in the Wenchuan earthquake-stricken area to suppress their instinctive choice of PS and HA. Meanwhile, it also can be seen that many residents in the secondary geological disaster area have the following ambivalence: attachment to their current place of residence, embarrassment, fear of new surroundings, and unknown interpersonal relationships are intertwined (67). As a result, the inner world is full of butterflies, and these complex psychological feelings will largely influence the residents' willingness to migrate and their consciousness of PS and HA.

For the advancement and insufficiency of this paper, first of all, the data in this paper is the first-hand information obtained by spending several months on field research, which is original. Then, the limitation of this paper is that the influencing factors are not comprehensive enough. With the results of field surveys and previous studies, this paper explores the influence of gender, education level, household income, the convenience of living, expectations for future life, and psychological feelings on residents' willingness to migrate. Yet, these are not enough, and many other factors affect residents' desire to relocate, such as the frequency of secondary geological disasters and their negative impacts.

Therefore, there are two suggestions for future research. Firstly, scholars should study residents' willingness to migrate from the perspective of geological hazards. Secondly, the research on residents' willingness to relocate should be improved by further refining the influencing factors.

## Conclusion and policy recommendations

This paper used 957 effective survey samples from 12 towns in the Wenchuan earthquake-stricken area to study the willingness of residents to migrate from the secondary geological disaster areas and their influencing factors. The results show that: (1) Faced with the unfavorable conditions of frequent secondary geological disasters, 54.8% of the residents in the disaster areas do not make the inevitable choice of PS and HA. (2) Whether residents migrate or not is affected by disasters, life convenience, family income, expectations for future life, gender, education level, psychological feeling, and other factors. (3) The lower the living convenience, the lower the family income, and the lower the education level, the more pessimistic the residents are about their future life and the more inclined they are to migrate. In terms of gender, female is more willing to migrate. (4) There is an inverse relationship between psychological feeling and migration intention. It means that when faced with frequent secondary geological disasters, residents are still willing to migrate under the principle of disaster avoidance, even if they have a strong attachment to the place they live in.

The study results have important implications for enriching population migration studies at the micro-level. The questionnaire survey and specific analysis of the secondary geological disaster areas in the Wenchuan earthquake-stricken areas are conducive to improving the wellbeing of residents. At the same time, the results are also specific and universal, and the proposed policy recommendations can provide references for the migration of residents and the reconstruction of the social governance system in secondary geological disaster areas.

After the reconstruction of the Wenchuan earthquake-stricken area, the residents are still faced with numerous secondary geological disasters that greatly threaten their lives. When suffering from secondary geological disasters such as landslides and debris flows directly threatening life safety, some residents in the disaster areas still have to migrate. The government should carry out the work according to the residents' willingness to move, combined with the reality, and enhance the residents' awareness and ability of PS and HA. In principle, most affected people are willing to migrate after being threatened by secondary geological disasters. However, their willingness to migrate or relocate is also different due to different living conveniences in different places, different family economic conditions, expectations for future life, education levels, and psychological feelings. Based on this, this paper proposes the following suggestions from the early, interim, and late stages of migration:

- Before the migration. The residents' family conditions and educational levels varied. Therefore, residents' property loss and their ability to PS and HA should be considered. It ensures that there is no significant difference in the living conditions of families before and after the migration. At the same time, the government should actively develop employment opportunities, explore more employment methods, and encourage people with higher education levels to work in local areas. Vocational skills training should be actively carried out to enable people with less education to do their best and appropriately raise the subsidy standard for local talents to reduce their outflow.
- During the migration process. More attention should be paid to the change in residents' ideology rather than tough relocation. The government should be patient with residents deeply attached to their hometown. At the same time, the site selection of the migration location should also consider the security of residence. The government should build secondary geological disaster protection projects in the resettlement areas and maintain good public order so that residents can live and work better after migration.
- After the migration. The most crucial point for the government is to consider whether the living convenience of residents has been improved or not. The building of health care systems, transportation, and other infrastructures should be strengthened to make people's life more convenient. Residents should feel that their life will be more suitable after migration, raising their expectations for the future.

## Data availability statement

The original contributions presented in the study are included in the article/supplementary material, further inquiries can be directed to the corresponding author.

## Author contributions

HH: funding acquisition, project administration, and resources. FW: methodology, visualization, roles/writing-original draft, writing–review and editing, software, and validation. YX: investigation, software, validation, conceptualization, data curation, and supervision. YL, H-LZ, and JC: writing-review and editing. All authors contributed to the article and approved the submitted version.

## Funding

This work was supported by the National Natural Science Foundation of China (41790445); Science and Technology Think Tank Research Topics of Sichuan Association for Science and Technology (sckxkjzk2021-4); Social Science Planning Major Project of Sichuan Province, China (SC20ZDCY001); Social Science Planning Major Cultivation Project of Chengdu University of Technology, China (YJ2021-XP001); Key Project of Resource Based Cities Development Research Center of Panzhihua University, China (ZYZX-ZD-2001).

## Conflict of interest

The authors declare that the research was conducted in the absence of any commercial or financial relationships that could be construed as a potential conflict of interest.

## Publisher's note

All claims expressed in this article are solely those of the authors and do not necessarily represent those of their affiliated organizations, or those of the publisher, the editors and the reviewers. Any product that may be evaluated in this article, or claim that may be made by its manufacturer, is not guaranteed or endorsed by the publisher.

## Abbreviations

PS: Profit-seeking
HA: Harm-avoiding
LPM: linear probability model.
